# A grazing-driven positive nutrient feedback loop and active sexual reproduction underpin widespread *Noctiluca* green tides

**DOI:** 10.1038/s43705-022-00187-4

**Published:** 2022-10-20

**Authors:** Hao Luo, Jingtian Wang, Joaquim I. Goes, Helga do R. Gomes, Khalid Al-Hashmi, Craig Tobias, Claudia Koerting, Senjie Lin

**Affiliations:** 1 State Key Laboratory of Marine Environmental Science, College of the Environment and Ecology, and College of Ocean and Earth Sciences, Xiamen University, 361102 Xiamen, China; 2 Lamont-Doherty Earth Observatory at Columbia University, Palisades, NY 10964 USA; 3 Department of Marine Sciences and Fisheries, Sultan Qaboos University, Muscat, Oman; 4 Department of Marine Sciences, University of Connecticut, Groton, CT 06340 USA

**Keywords:** Environmental sciences, Water microbiology

## Abstract

The mixoplankton green *Noctiluca scintillans* (g*Noctiluca*) is known to form extensive green tides in tropical coastal ecosystems prone to eutrophication. In the Arabian Sea, their recent appearance and annual recurrence have upended an ecosystem that was once exclusively dominated by diatoms. Despite evidence of strong links to eutrophication, hypoxia and warming, the mechanisms underlying outbreaks of this mixoplanktonic dinoflagellate remain uncertain. Here we have used eco-physiological measurements and transcriptomic profiling to ascribe g*Noctiluca*’s explosive growth during bloom formation to the form of sexual reproduction that produces numerous gametes. Rapid growth of g*Noctiluca* coincided with active ammonium and phosphate release from g*Noctiluca* cells, which exhibited high transcriptional activity of phagocytosis and metabolism generating ammonium. This grazing-driven nutrient flow ostensibly promotes the growth of phytoplankton as prey and offers positive support successively for bloom formation and maintenance. We also provide the first evidence that the host g*Noctiluca* cell could be manipulating growth of its endosymbiont population in order to exploit their photosynthetic products and meet critical energy needs. These findings illuminate g*Noctiluca*’s little known nutritional and reproductive strategies that facilitate its ability to form intense and expansive g*Noctiluca* blooms to the detriment of regional water, food and the socio-economic security in several tropical countries.

## Introduction

Green *Noctiluca scintillans* (hereinafter g*Noctiluca*) is one of the fastest biogeographically spreading marine planktonic organisms in the world’s oceans [1–5]. The dinoflagellate *N. scintillans* is an enigmatic species because it has the capability to occupy exclusively heterotrophic (red *Noctiluca*, r*Noctiluca*) as well as mixoplanktonic niches (g*Noctiluca*) [4]. The photoautotrophy exhibited by g*Noctiluca* is due to a seemingly loose but exclusive mutualistic partnership with the prasinophyte green alga *Protoeuglena noctilucae* [6–8]. This exclusive relationship between the host and free-swimming endosymbionts makes g*Noctiluca* different from other mixoplankton that have either permanent chloroplasts, or those that sequester (steal) chloroplasts (kleptoplasty). g*Noctiluca* also feeds on other protists especially diatoms as evidenced in the Arabian Sea [9] and in the Gulf of Thailand [10] as well as in laboratory experiments [11–13]. Conversely, the more cosmopolitan r*Noctiluca*, which is thought to acquire an orange-red coloration because of carotenoids from the plankton it feeds on [14], is exclusively heterotrophic, has no symbionts and is a voracious grazer [15–17]. Unlike r*Noctiluca*, which is globally ubiquitous [4, 18], g*Noctiluca* is restricted to the tropics and sub-tropics and particularly in coastal ecosystems that are subjected to protracted nutrient overloading and hypoxia, either due to domestic sewage outflow, or heavy use of synthetic fertilizers for agriculture [2, 9, 17, 19].

g*Noctiluca’s* emergence as a dominant bloom forming organism in the Arabian Sea during the winter monsoon is fairly recent [3, 5, 20–23] and no reports exist of these blooms in the northern Arabian Sea prior to 2000. Their sudden appearance has been linked to the intrusion of sub-oxic waters from an expanding Oxygen Minimum Zone [9, 24], which stimulates endosymbiont photosynthesis and changes in inorganic nutrient stoichiometry [25]. In addition, nutrient replenishment of oligotrophic waters from winter convective mixing [26] produces large diatom blooms [27–30] that serve as a food source for this mixoplankton. In the Sea of Oman, g*Noctiluca* blooms were also first seen around 1999 [31] and since then, have been appearing almost year round, and often, in association with the shoaling of hypoxic water [3, 32–34].

In the Arabian Sea, it is still inexplicable how *gNoctiluca* blooms, which first appear in late December and early January, are able to persist even until late March when waters turn oligotrophic, before their termination either by grazing by its major predators, salps and jellyfish, and/or lethal rising seawater temperatures [9, 35]. Along the coast of Oman, g*Noctiluca* alone or in combination with salps and jellyfish swarms are especially devastating to artisanal fisheries and freshwater supply from desalination plants [36]. While clearly there have been considerable advances in our understanding of the ecology of this organism, very little is known about the molecular and metabolic mechanisms that underpin its capacity to outgrow other phytoplankton and mixoplankton to form massive green tides. Several studies have examined the formation of *Noctiluca* blooms in various ecosystems, but most have focused on the red form (r*Noctiluca*) [1, 17, 20]. g*Noctiluca* on the other hand, is less studied, with its fundamental nutrition and reproduction mechanisms remaining elusive. Here we report findings from a field study that integrated ecological, physiological, and molecular analyses during g*Noctiluca* blooms in the Sea of Oman, which provide novel insights into its fundamental reproductive and nutritional strategies that contribute not only to the formation of g*Noctiluca* blooms but to their persistence even under conditions unfavorable to most other protists.

## Materials and methods

### Study areas, sample collection, species isolation and measurements

Our primary sampling site was Seeb Jetty, located in a shallow semi-enclosed bay in Muscat, Oman (Fig. S1), chosen because of its proximity to the shore laboratory, and because g*Noctiluca* blooms occur here with predictable regularity every year. We also sampled at 6 stations (ST0 to ST5) along a transect that extended 20 km offshore into the Sea of Oman (Fig. S1). Sampling offshore was guided by Level 2 Aqua-MODIS satellite images of the previous day, which showed high Chl *a* concentrations (>10 mg m^−3^) and blooms in offshore flowing filaments associated with a cyclonic eddy (Fig. S1). Sampling at Seeb Jetty was undertaken daily from the 2nd to 11th Feb. 2018, usually at high tide each day, to monitor the development of the g*Noctiluca* bloom.

After recording surface seawater temperatures, 10 L of surface seawater were collected in a polycarbonate carboy that had been previously washed with 5% technical grade HCl and rinsed with deionized water. Water samples were immediately covered with a black sheet to shield the biological constituents from light, and immediately transported to the laboratory for further analysis as described below.

In the laboratory, the carboy was gently mixed, and duplicate samples of 50 mL were immediately fixed with two drops of glutaraldehyde for microscopy and FlowCAM [12] determination of cell counts and phytoplankton taxonomy. Glutaraldehyde was chosen over buffered Formalin + Lugol’s iodine as it preserves the g*Noctiluca* cells intact. Aliquots of 50–300 mL of seawater sample were collected daily, and filtered onto 0.7 µm Whatman GF/F. Filters were stored at −20 °C for measurements of phytoplankton Chl *a* extracted from the filters in cold 90% acetone and fluorometrically determined in a Turner Designs Trilogy fluorometer pre-calibrated with standard Chl *a* (Sigma-Aldrich). Phaeopigments were estimated by acidifying the sample with 1 M HCl.

For analysis of dissolved nutrients (nitrite, nitrate, ammonium and dissolved inorganic phosphate), 50 mL of seawater was gently filtered through a 0.22 µm Nucleopore syringe filter and stored at −20 °C until analysis in a SmartChem (Westborough, MA, USA) discreet nutrient auto-analyzer. Dissolved inorganic phosphorus measurements were determined by colorimetric method for orthophosphate using the USEPA method 365.1. Nitrogen (nitrate, nitrite, ammonium and urea) measurements were based on American Public Health Association’s protocols.

Spearman’s rank correlation coefficient (corrplot library in R) [37] was undertaken to infer the relationships between nutrients (nitrate, nitrite, ammonium and phosphate), cell counts and Chl *a* in two phytoplankton fractions (whole and <200 µm).

### Growth rates of *P. noctilucae* in symbiosis with g*Noctiluca* and as a free-living culture

These experiments were undertaken in the laboratory using isolates of *gNoctiluca* with its symbiont *P. noctilucae* and as well as *P. noctilucae* isolated from *gNoctiluca* cells and cultured independent of the host. About 2000 *gNoctiluca* cells growing exponentially in f/20 media, were transferred into a set of triplicate 2 L polycarbonate bottles. Ten mL of exponentially growing cells of *P. noctilucae* were transferred into another set of triplicate 2 L polycarbonate bottles containing f/20 media. Both sets of bottles were incubated for 10 days under identical conditions at 26.5 °C under a light:dark cycle of 14 L:10 D with a photon flux of about 200 µmol photons m^−2^ s^−1^. Growth rates were based on changes in Chl *a* concentration recorded during the mid-exponential phase of cells for each flask.

### Sample collection, RNA isolation, and high-throughput metatranscriptome sequencing

For transcriptomic analyses, duplicate field samples (collected on 2nd Feb.) were filtered onto Nucleopore 3 µm pore size polycarbonate filters, immediately fixed in 1 mL Trizol and transferred into liquid Nitrogen. In parallel, to profile gene expression in the aposymbiotic alga *P. noctilucae* growing in the free-living mode, 50 mL (~1 × 10^6^ cells) aliquots of *P. noctilucae* were filtered onto 3 µm Nucleopore membrane filters, and transferred into a 2 mL microcentrifuge tube containing 1 mL Trizol and immediately placed in liquid nitrogen.

To isolate RNA, both sets of samples were disrupted using a FastPrep-24 bead mill (MP Biomedicals, Solon, OH, United States) prior to RNA extraction as previously reported for field samples [38]. Total RNA was extracted following protocols detailed in Lin et al. [39] and Luo et al. [40] and quantified using a NanoDrop-2000 Spectrophotometer (Thermo Fisher Scientific, United States). For RNA high-throughput sequencing, 1 µg of total RNA was used for mRNA enrichment using NEBNext Poly (A) mRNA Magnetic Isolation Module (New England Biolabs, United States). mRNA was then fragmented and used as template to synthesize double-strand cDNA. Fragments with specific size (~400 bp) were chosen for sequencing on an Illumina HiSeq 4000 instrument according to the protocol of Zhang et al. (2019) [38].

### Bioinformatic analysis

Raw reads were trimmed to eliminate low quality reads and reads with adaptor using Trimmomatic V0.30 [41]. The resultant clean reads were assembled *de novo* in Trinity 2.0, and clustered to unigenes using TGICL (TIGR Gene Indices clustering tools, V2.1) [42]. These unigenes were annotated against NCBI non-redundant (nr) database, Swiss-Prot and Pfam database with a cutoff of 1e−5 and mapped to KEGG pathways for further analysis. Meanwhile, to quantify the expression level of genes in g*Noctiluca*, clean reads were mapped to the *Noctiluca* database in MMETSP0253 using Bowtie2 (parameters: -sensitive). Gene expression was quantified by normalizing the mapped reads to total transcriptomic reads of g*Noctiluca* and presented as FPKM value (Fragments Per Kilobases per Million mapped reads). Most highly expressed genes (HEGs) (top 25%) were further examined for their functions potentially related to bloom development.

### Genes differentially expressed between g*Noctiluca* bloom and cultured r*Noctiluca*

Besides the analysis of most HEGs, we also investigated the genes that were upregulated during bloom development. This would be ideally undertaken by comparing the bloom samples with pre-bloom samples. Unfortunately, our pre-bloom samples contained too few g*Noctiluca* cells to obtain meaningful data (most g*Noctiluca* genes showed zero expression level). As a compromise, we compared the bloom samples with cultured *Noctiluca* samples (MMETSP), assuming that the culture condition was not quite a bloom condition. Differentially expressed genes (DEGs) between our metatranscriptome (OMF02) and transcriptome data (SRR1296929) were analyzed using edgeR V3.28.1 [43]. The moderate biological coefficient of variation of 0.2 was used to estimate dispersion. DEGs of edgeR were screened with a threshold of FDR ≤ 0.05 and the log2(Fold change) ≥1.

### Nutrient release experiments

g*Noctiluca* cells were collected from blooms at two locations, one at Seeb Jetty on 3rd Feb. 2018 and the second from ST0 on 13th Feb. 2018. For these and the following experiments, all 500 mL clear polycarbonate bottles were acid-washed and soaked overnight with 5% laboratory grade HCl, rinsed with Milli-Q distilled water, and autoclaved prior to use.

For each experiment, 500 mL aliquots of whole water samples were gently pre-filtered through a sterilized 200 µm mesh net. The filtrate was discarded and the g*Noctiluca* cells retained on the net were gently washed with 0.22 µm pre-filtered seawater (FSW) from the study site to eliminate other species, and then carefully transferred into 4 × 500 mL polycarbonate bottles containing FSW. After sampling for initial nutrient (urea, ammonium, nitrate, nitrite and phosphate) concentrations, 2 bottles were covered with four layers of aluminum foil (dark treatment), while 2 were left uncovered (light treatment). All 4 bottles were incubated under ~70 µmol photons m^−2^ s^−1^, and a photoperiod of 14 L:10 D, for 8 days at 26.5 °C without adding nutrients or prey. 25 mL aliquots were drawn from each bottle for the first 2 days and every second day over the next 5 days. The aliquots were gently filtered through a 0.22 µm Nucleopore syringe filter and the filtrate was stored at −20 °C for nutrient analysis in the SmartChem auto-analyzer as described above.

### Nitrogenous nutrient (ammonium and urea) and carbon uptake experiments

Nitrogenous and carbon uptake experiments were also undertaken on seawater samples from the Seeb Jetty on 3rd and 7th Feb as well as from samples collected from the 6 offshore stations ST0- ST5. The experiments were conducted using whole seawater samples, which included g*Noctiluca* and phytoplankton, and samples pre-filtered through a 200 µm Nybolt mesh to remove g*Noctiluca*. Microscopic examination showed this fraction (<200 µm fraction) consisted mostly of diatoms but also some small-sized g*Noctiluca* that may have passed through the mesh. Any large grazers visible in the whole water sample were removed with a plastic Pasteur pipette before the sample was transferred into bottles. Prior to start of the experiment, separate 50 mL aliquots of whole water samples were syringe filtered (0.22 µm Nucleopore) and the filtrate was stored at −20 °C for nutrient analyses.

The two fractions (whole water and <200 µm) were then dispersed into triplicate 250 mL acid-washed, Milli-Q water rinsed and autoclaved polycarbonate bottles. The bottles were amended with ^15^N labeled NH_4_Cl (98% atom ^15^N) such that final ^15^N-NH_4_ concentrations were 1 µM_._ A similar set was spiked separately with 1 µM ^15^N labelled urea (98% atom ^15^N). A third set was spiked with 1 µM ^13^C labelled bicarbonate (98% atom ^13^C). All bottles were sealed and placed in the incubator under same conditions as the nutrient release experiments described above. We were not able to conduct experiments with ^15^N labeled nitrate because it was unavailable at the time of the experiment.

After 24 h of the incubation, samples were gently filtered onto pre-combusted (400 °C for 1 h) Whatman GF/F filters, washed repeatedly with FSW to rid the filters of residual non-assimilated ^15^N nitrogenous nutrients and 13 C bicarbonate, dried in an oven at 60 °C, and stored in a desiccator until the time of analysis. Nitrogen and carbon content as well as ^15^N and 13 C isotopic enrichment on the filters were measured on a Thermo Delta V Plus continuous flow stable isotope ratio mass spectrometer equipped with a Costech 4050 Elemental Analyzer [44]. Nitrogen and carbon uptake rates were calculated following standard protocols [45] after correcting for residual isotope on the filters from the control samples.

Nitrogenous and carbon uptake rates of g*Noctiluca* were estimated by subtracting the uptake rates of the <200 µm fraction from that of the whole seawater samples. The difference represented the lower bound of g*Noctiluca* uptake rates, because some small-sized g*Noctiluca* passed into the <200 µm fraction, with the rates in the whole water being the upper bound.

## Results

### g*Noctiluca* bloom dynamics

**Fig. 1:**
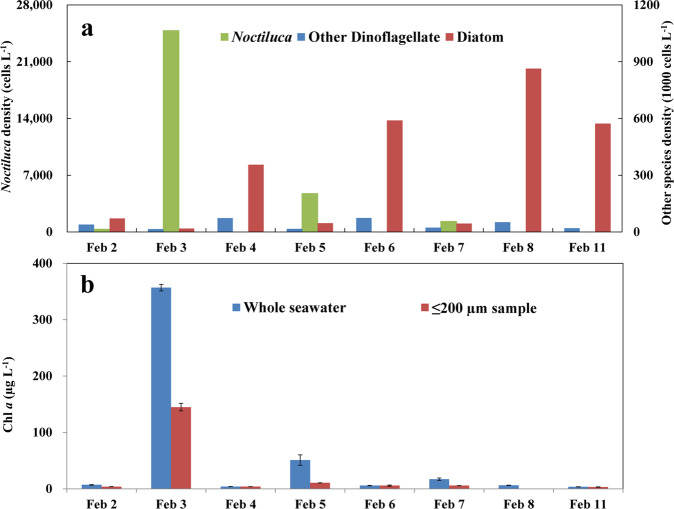
g*Noctiluca* and phytoplankton dynamics in the study period during a bloom at Seeb Jetty, Muscat, Sultanate of Oman. **a** Contrasting cell abundances (cells L^−1^) between g*Noctiluca* and phytoplankton functional groups. **b** Chlorophyll *a* (Chl *a*) concentration (µg L^−1^) for whole seawater and ≤200 µm size fraction (excluding most g*Noctiluca* cells). Error bar stands for standard deviation.

The study was undertaken from 2nd Feb. to 11th Feb., 2018 at Seeb Jetty, Muscat, a sheltered bay along the coast of the Sultanate of Oman where g*Noctiluca* was in an early stage of bloom formation (Fig. S1). After a slow start, g*Noctiluca* cell densities rose from 3.9 × 10^2^ cells L^−1^ on 2nd Feb. to 2.5 × 10^4^ cells L^−1^ on the following day (Fig. 1a), resulting in a bloom with a high cell-specific growth rate of 1.86 d^−1^ that dramatically changed the color of the previously clear bay waters to green. On the next day (4th Feb.), the color of the waters abruptly changed to golden yellow, and g*Noctiluca* cell numbers dropped to almost negligible. However, on 5th Feb., the waters turned light green and g*Noctiluca* cell concentrations rose once again with cell counts of 4.8 × 10^3^ cells L^−1^. This boom-and-bust cycle repeated itself on the 7th Feb. although the cell numbers were lower (1.3 × 10^3^ cells L^−1^), following which g*Noctiluca* counts remained low until their reappearance in small numbers on the 11th of Feb. (28 cells L^−1^). Microscopy and FlowCAM analyses also showed the presence of diatoms and dinoflagellates, but cell counts of both groups were always very low when g*Noctiluca* counts were high (Fig. 1a). Diatom counts were highest on the 8th of Feb. (8.6 × 10^5^ cells L^−1^), likely due to reduced grazing pressure from g*Noctiluca* and a sudden increase in nutrients in the water column from death and decay of g*Noctiluca* cells (Fig. S2a). Chl *a* concentrations in whole water samples exhibited a strong positive correlation with g*Noctiluca* abundance (Fig. 1b; Fig. S3) indicating a healthy population of photosynthesizing symbionts, *P. noctilucae*, in the host cell. Throughout the study period, seawater temperature was fairly stable (22.5 ± 0.3 °C), excluding the contribution of temperature to the bloom dynamics.

### Active sexual reproduction during bloom development

*Noctiluca* is known to reproduce asexually and sexually, and a high number of gametes observed in the host cells (progametes) suggest a potentially vital role of sexual reproduction as an accelerator for bloom formation [10, 46–49]. We also observed gametogenesis inside g*Noctiluca*, and zoospores (by morphology mixture of zygotes and released mature gametes) were especially abundant when the bloom transitioned from green to golden yellow (Figs. S4 and S5). Because it is very challenging to distinguish zygotes from free-swimming gametes in preserved samples, counting the abundance of zygotes was not possible. We used an indirect method, i.e., estimating the percentage of g*Noctiluca* cells that were in gametogenesis and multiplying it by the number of gametes each gametocyte produces previously reported [10] for *Noctiluca*, then dividing the value by two (two gametes produces one zygote). We observed that the percentages of g*Noctiluca* experiencing sexual reproduction on Feb 2, Feb 4, and Feb 6 were clearly more than that on Feb 3, Feb 5, and Feb 7 (Fig. S6), when the peak of g*Noctiluca* density appeared. We estimated that ~50% of g*Noctiluca* carried progametes on Feb 2, and according to previous reports of ~1024 gametes produced by one *Noctiluca* gametocyte, this potentially gave a maximum of 256 doubling of cells per day, which roughly agrees with our observed growth rate described above.

**Fig. 2:**
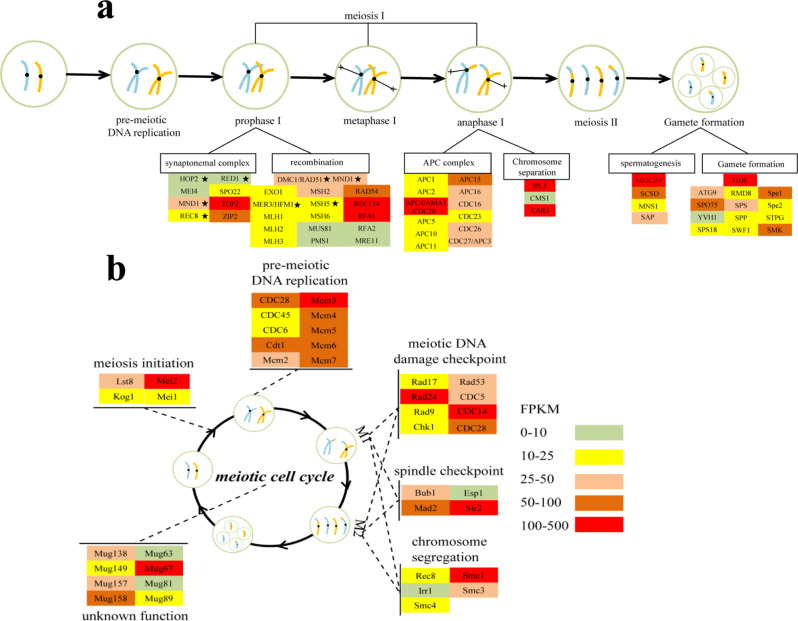
Active sexual reproduction in g*Noctiluca* during the bloom. **a** Expression profile of genes associated with major events in the gametogenesis process in g*Noctiluca*. **b** Expression profile of meiosis regulatory genes. Asterisks depict eukaryotic meiosis-specific core genes. Color bar and scale in **a** denotes gene expression level (normalized by FPKM), which is also applicable to **b**.

Consistent with our field observations of zygotes, transcriptomic data of g*Noctiluca* from Seeb Jetty on 2nd Feb., immediately before the bloom, showed 97 meiosis-related genes (Fig. 2), most of which were HEGs. As our metatranscriptomics sequences were annotated against previously reported *Noctiluca* transcriptome data (MMETSP0253; see Materials and Methods) with strict parameter setting, these genes (and all others discussed in this paper) were clearly from g*Noctiluca*. Further analyses indicated that seven of them were eukaryotic meiosis-specific genes: *HOP2*, *MND1*, *REC8*, *RED1*, *DMC1*, *MER3/HFM1* and *MSH* [50–52]. Among these, *HOP2* and *MND1* interact with *DMC1* by constituting a heterodimeric complex [53], promoting the interhomolog meiotic recombination [54]. REC8 is a meiosis-specific cohesin subunit [55] and RED1 is a key meiotic prophase factor [56], both known to play an important role in meiosis I in typical sexual organisms. MER3/HFM1 and MSH4/MSH5 are components of ZMM proteins (also known as synapsis initiation complex) that directly promote meiotic recombination (Fig. 2a) [57]. Some other meiosis-specific genes documented in model sexual eukaryotes, such as *HOP1*, *MSH4*, *SPO11* and *ZIP1*, were not detected, probably because g*Noctiluca* does not use these genes or has evolutionarily replaced them with alternatives. For instance, genes MRE11 and EXO1 that we identified in g*Noctiluca* are known to perform the same function as SPO11, which is to repair meiotic DNA double-strand breaks (DSBs) [58].

Also represented in the transcriptomes was the anaphase-promoting complex (APC) (Fig. 2a), which is important in both meiotic and mitotic cell cycles as it induces the degradation of several factors that act on spindle-pole separation and spindle disassembly [59]. Among the several subunits of APC detected, APC15/MND2 is essential to meiosis but not required for mitosis [60]. We also identified *KAR3* and *IPL1*, which are vital for homologous chromosome synapsis during meiosis [61, 62], both of which were highly expressed (i.e., in the HEGs group).

Furthermore, transcriptomes contained many genes associated with gamete formation such as *MMCAP* that functions in spermatogenesis [63], as well as *MNS1*, *SAP* and *SCSD*. Interestingly, several of these genes (*SPE*, *STPG*, *ODF*, *SPO75*, *SPS18*, *RMD8*, *SPS* and *SWF1*) are known to be involved in the structure of sperm flagella and sporulation in mice [64, 65] and yeast [66, 67]. We also identified a series of meiosis regulatory genes (Fig. 2b), of which, Mei2, was highly expressed (FPKM = 190.03). Mei2 is a regulatory RNA-binding protein which plays a vital role in the meiosis initiation [68, 69] and was originally discovered in yeast, then in plants, and more recently in several algal species including *Fugacium kawagutii* [70], *Scrippsiella trochoidea* [71], *Prorocentrum donghaiense* [38], *Heterosigma akashiwo* [72], and *Prorocentrum cordatum* [73]. Other highly expressed meiosis genes found in the g*Noctiluca* bloom included mini chromosome maintenance proteins (MCM2-7), which plays an important role in DNA replication during pre-meiotic S phase [74]. *CDC14* and *CDC28*, *Mad2*, *Rad24* and *Smc1*, which regulate M phase, spindle checkpoint and DNA damage checkpoint, respectively [67]. Finally, several meiotically up-regulated genes, originally described in yeast (*Mug*, Fig. 2b), were also actively expressed in the g*Noctiluca* bloom.

Moreover, we also compared the expression level of some meiosis genes in our bloom samples with that in the previously reported r*Noctiluca* laboratory culture samples (MMETSP0253). Among the genes common in the two sets of samples, four meiosis-specific genes (*MND1*, *REC8*, *DMC1* and *MSH5*) were more highly expressed in our sample than in the cultured sample, and some meiosis-related genes such as Mei2 also exhibited significantly higher expression (*P* < 0.05) (Fig. S7). These above findings clearly demonstrate the active sexual reproduction (meiosis to produce gametes) during the *gNoctiluca* bloom, suggesting its vital role in driving the bloom.

### Positive nutrient feedback loop associated with the bloom

It is generally believed that mixoplankton turn to phagotrophy when nutrients are depleted in the environment [7]. During our study period, P and N nutrient concentrations at Seeb Jetty were non-limiting and showed a striking increase. Urea, ammonium, nitrate and phosphate concentrations were moderate (3.5, 2.5, 2.8 and 0.74 µM respectively) on 2nd Feb., but rose by approximately tenfold (34.1, 32.8, 30.9 and 18.2 µM, respectively) on 3rd Feb. (Fig. S2a), when the g*Noctiluca* bloom appeared (Fig. 1a). Similarly, at the sampling station ST0 in the coastal waters of the Sea of Oman, where the bloom was also observed, urea, ammonium, nitrate and phosphate concentrations were also high (13.23, 17.55, 3.01 and 8.68 µM) (Fig. S2b). Nutrients at non-bloom sites remained at moderate levels. Thus, the elevated nutrient concentrations in bloom waters were most likely from g*Noctiluca*.

With previous information that r*Noctiluca* and g*Noctiluca* accumulate high concentrations of ammonium and urea within their central cytoplasm [12, 75], we conducted experiments to examine if g*Noctiluca* was the source of the nutrients measured in the water. When g*Noctiluca* cells from two separate blooms were incubated in 0.22 µm-filtered seawater in light and dark bottles for 5–8 days without addition of nutrient or prey, we measured steady increases of nutrients in the media during the incubation. Within a day, nutrients increased in both treatments by 132–281 µM for ammonium, 23.6–50.3 µM for urea, 2.5–18.1 µM for nitrate and 39.4–68.1 µM for phosphate (Fig. S8a). Of the total released nitrogenous nutrients (sum of nitrite, nitrate, ammonium, and urea), ammonium accounted for >78% (Fig. S8b). For such an enormous amount of ammonium to be released, it firstly had to accumulate within g*Noctiluca*, either via active cell membrane transport and/or ammonium regeneration from digesting and metabolizing of ingested prey.

**Fig. 3:**
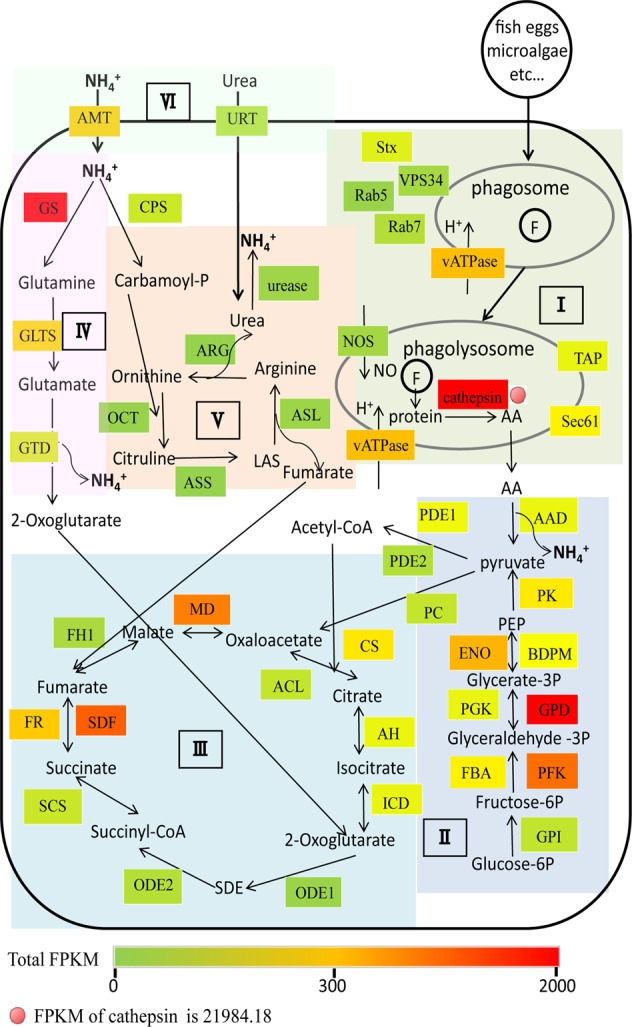
Highly expressed genes (top 25%) and their related metabolic processes in g*Noctiluca*. **I** Phagocytosis metabolism. **II** Glycolysis. **III** TCA cycle. **IV** Ammonium metabolism. **V** Urea cycle. **VI** Nitrogen transport. Color bar denotes gene expression level (normalized by FPKM). Note that the expression of cathepsin was so high (FPKM = 21984.18) that it far exceeds the color scale used to properly indicate the differential expression of other genes. Stx Syntaxin, Rab5 Ras-related protein Rab5, VPS34 Phosphatidylinositol, 3-kinase vps34, vATPase V-type proton ATPase, Rab7 Ras-related protein Rab7, NOS Nitric oxide synthase, Sec61 Protein transport protein Sec61, TAP ABC transporter B family, AAD amino acid deaminase, GPI Glucose-6-phosphate isomerase, PFK Phosphofructokinase, FBA Fructose-bisphosphate aldolase, GPD Glyceraldehyde-3-phosphate dehydrogenase, PGK Phosphoglycerate kinase, BDPM 2,3-bisphosphoglycerate-dependent phosphoglycerate mutase, ENO Enolase, PK Pyruvate kinase, PDE1 Pyruvate dehydrogenase E1 component, PDE2 Pyruvate dehydrogenase E2 component, PC Pyruvate carboxylase, CS Citrate synthase, ACL ATP-citrate (pro-S-)-lyase, AH Aconitate hydratase, ICD Isocitrate dehydrogenase [NADP], ODE1 2-oxoglutarate dehydrogenase complex component E1, ODE2 2-oxoglutarate dehydrogenase complex component E2, SCS Succinyl-CoA synthetase, FR Fumarate reductase, SDF Succinate dehydrogenase [ubiquinone] flavoprotein subunit, FH1 Fumarate hydratase class I, MD Malate dehydrogenase, AMT Ammonium transporter, GS Glutamine synthetase, GLTS Glutamate synthase, GTD NAD-specific glutamate dehydrogenase, CPS Carbamoyl-phosphate synthase [ammonia], OCT Ornithine carbamoyltransferase, ASS Argininosuccinate synthase, ASL Argininosuccinate lyase, ARG Arginase.

Nitrogen uptake measurements of g*Noctiluca* with ^15^N labeled ammonium or ^15^N labeled urea (Fig. S9) clearly showed g*Noctiluca*’s N-nutrient uptake and its preference for ammonium over urea. We also saw higher uptake rates of ammonium and urea at bloom sites relative to non-bloom sites (Fig. S9). In accordance with the uptake data, both ammonium and urea transport genes (*AMT* and *URT* respectively) were actively expressed in g*Noctiluca*, but the expression of *AMT* (FPKM = 443.3) was four times higher than that of *URT* (FPKM = 101.1) (Fig. 3). Although we were unable to conduct parallel ^15^N-nitrate uptake incubations, nitrate transporter genes in g*Noctiluca* were absent in the transcriptome, implying lower preference for or inability to use nitrate as a nutrient source.

Our metatranscriptomes also indicated active grazing as a major source of accumulated N-nutrients in g*Noctiluca*. A large fraction of genes involved in phagocytosis and nitrogenous molecule metabolism was highly expressed (Table S1, Fig. 3). One of these genes encodes the digestive enzyme cathepsin, which cleaves proteins and peptides [76], potentially producing amino acids, and it was the most highly expressed gene family found in this study with a total FPKM of 21,984.18 (Fig. 3). Furthermore, the HEG pool also contained genes encoding amino acid deaminases (FPKM = 243.49) that act on amino acids to produce ammonium. In addition, NAD-specific glutamate dehydrogenase (FPKM = 196.30) and urease (FPKM = 36.6), catalyzing reactions that generate ammonium from glutamate and urea, respectively, were also highly expressed (Fig. 3).

Phagocytosis mainly consists of three processes, which together include motility, prey capture, phagosome maturation and prey digestion [77, 78]. Genes that encode actin and tubulin, the structural components of cytoskeleton, were most abundant and most highly expressed (*SI Appendix*, Table S1). This set of HEGs also included some motility-related genes that encode kinesin, myosin, dynein and flagella-related proteins. Also highly expressed in our transcriptome were *MRCK*, *ARP2/3* complex, *calreticulin* and *calnexin*, known to be associated with particle uptake [77, 79]. Of these, the *ARP2/3* complex and *calreticulin* were HEGs. Nine other HEGs involved in the maturation of phagosome, including small GTPase *Rab5* and *PIK3C3*, as well as *vATPase* that act to regulate phagosome acidification were also recorded. Syntaxin and Sec22 as parts of SNARE complex (soluble N-ethylmaleimide-sensitive factor activating protein receptor), which are responsible for endosome membrane fusion and vesicle trafficking, were also highly expressed. The two other HEGs, *TAP* and *Sec61*, are involved in regulation of transmembrane transport in phagolysosome. A range of HEGs involved in the production of ammonium by phagocytosis (Fig. 3) complemented the increased concentration of phaeopigments (chlorophyll digestion products) in the bloom samples (Fig. S10), indicating that grazing on phytoplankton was likely the dominant energy and nutrition source for g*Noctiluca* bloom formation and the dominant mode of ammonium accumulation within g*Noctiluca* cells.

It is believed that accumulation of ammonium within the symbiosome of g*Noctiluca* helps support photosynthesis of the endosymbiont *P. noctilucae* [2, 9], which is evident from ^13^C-HCO_3_ tracer uptake experiments (Fig. S9). However, we speculate that ammonium accumulation within g*Noctiluca* may stimulate sexual reproduction in *gNoctiluca*. Although for most dinoflagellates, environmental stresses such as nutrient deficiency and temperature are the most common triggers [49, 80], *Noctiluca* is clearly an exception because its active gametogenesis occurred during actively growing blooms. Furthermore, ammonium is indeed able to induce sexual reproduction and gamete formation in the diatom *Thalassiosira pseudonana*, as evidenced by elevated expression of meiosis genes, triggering sexual morphologies, including oogonia, auxospores and spermatogonia [81]. This is consistent with our observations in g*Noctiluca*, where we also observed high expression of gamete-forming genes and active gamete formation during the bloom in conjunction with an incredibly large amount of ammonium released by g*Noctiluca*. If verified experimentally, this finding will have profound implications in dinoflagellate bloom ecology.

### High photosynthetic activity but inactive cell division in endosymbionts *in-hospite*: potential host manipulation for photosynthates

**Fig. 4:**
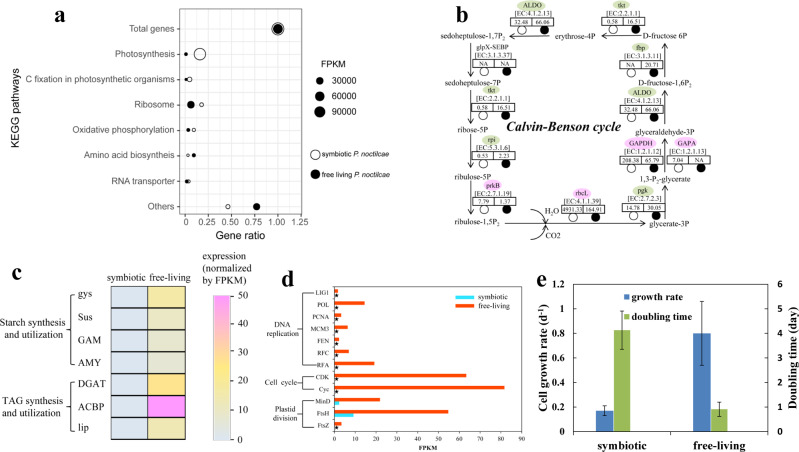
Transcriptomic proofs of host manipulation for photosynthates. **a** Enrichment of KEGG pathways in both *in-hospite* and free-living *P. noctilucae*, indicating photosynthesis was the most dominant metabolic activity in *in-hospite P. noctilucae*. **b** Expression of genes involved in Calvin-Benson cycle. Pink (green) denotes higher (lower) expression in *in-hospite P. noctilucae*. Expression level (FPKM) of each gene in both *in-hospite* and free-living *P. noctilucae* are indicated in the relevant boxes. **c** Low starch and TAG metabolisms in the *in-hospite P. noctilucae*. **d** Low cell division and relatively high plastid division in the *in-hospite P. noctilucae*. Asterisks depict the absence of genes in the metatranscriptomics data. **e** Low growth rate (d^−1^) and longer cell doubling time (days) of the *in-hospite P. noctilucae* compared to free-living counterparts under laboratory incubation. Error bar stands for standard deviation.

In mutualistic relationships between algae and animals (e.g., Symbiodiniaceae and Cnidaria), the host manipulates the nutrient supply of endosymbionts to maximize the production and translocation of photosynthates (organic carbon) and minimize symbionts’ reproduction [82–84]. In protist symbiosis, little has been explored about host manipulating symbionts except for enslaving kleptoplasts. However, in the *Acantharia*-*Phaeocystis* symbiosis, the symbiotic *Phaeocystis* were blocked from cell division and carbon storage, while their carbon uptake increased 150-fold and genes involved in photosynthesis and carbon fixation were upregulated, a clear case of host manipulating the symbiont [84]. Although, in the case of g*Noctiluca* and *P. noctilucae*, the exact mode of the mutualistic interactions is still unclear, our transcriptomic data showed that light reactions of endosymbiont photosynthesis were extremely active during the bloom, accounting for 89% of FPKM of all mapped genes (Fig. 4a). RbcL and GAPA, both critical genes for photosynthetic carbon fixation (dark reactions), were also highly expressed (Fig. 4b). This enhanced carbon fixation and carbon storage typically benefits the algae, but could also benefit the host. Intriguingly, biosynthesis and degradation of carbon storage substances such as triacylglycerol (TAG) and starch were transcriptionally inactive in the symbiotic alga (Fig. 4c), suggesting that most of the fixed carbon was not converted into lipid storage in the symbionts. Furthermore, genes related to DNA replication and cell division were markedly less expressed in the *P. noctilucae* living inside g*Noctiluca* as compared to free-living counterparts (Fig. 4d), consistent with the possibility that the host depresses symbiont population growth. Strikingly, results from our physiological experiments also revealed significantly lower growth rates for *P. noctilucae in-hospite* as compared to its free-living counterparts (Fig. 4e). In contrast, genes involved in plastid division, which is generally synchronized with cell division in unicellular algae, exhibited higher expression relative to the cell division genes (Fig. 4d). Given that plastid proliferation can enhance photosynthesis efficiency [85], this result agrees with the idea that g*Noctiluca* promotes photosynthesis of *P. noctilucae in-hospite*, but slows their reproduction. This would benefit the host by gaining an additional energy source to supplement that from grazing especially during the bloom when a large amount of energy is required for the explosive proliferation of g*Noctiluca*.

## Discussion

**Fig. 5:**
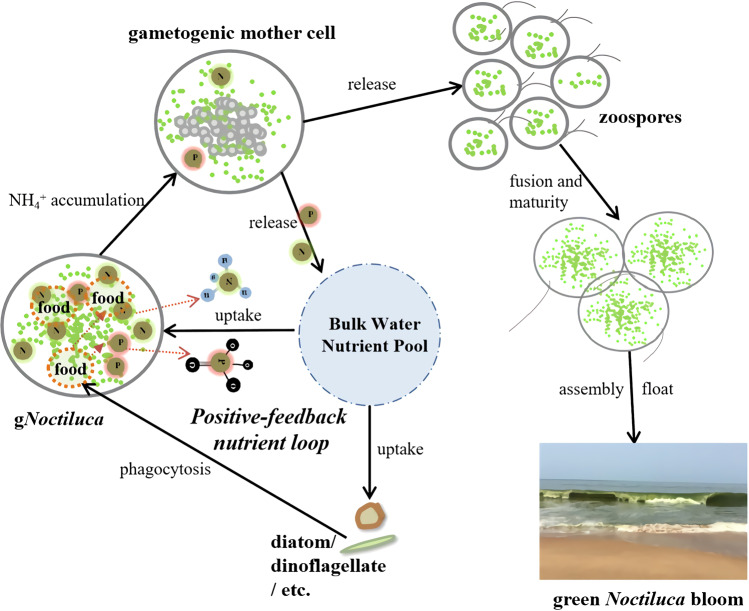
Schematic of a “positive feedback loop” nutrition strategy and sexual reproduction that promote the formation and maintenance of g*Noctiluca* blooms. The process by which nutrients first accumulate in *gNoctiluca* cells primarily via grazing, and are then released into the ambient environment during the sexual reproduction phase, to be then used by diatoms or dinoflagellates and ultimately back to support the growth of *gNoctiluca*, constitutes a positive feedback nutrient loop. Accumulation of ammonium may potentially stimulate the initiation of sexual reproduction of g*Noctiluca*, contributing to the outbreak of bloom.

Emerging from the present study is a sketch of a hitherto poorly understood process and mechanisms underpinning the rise of a g*Noctiluca* bloom (Fig. 5). It begins with nutrients (N and P), which are essential for algal growth and the formation of algal blooms [86–88]. Our Spearman correlation analysis showed significant positive correlations (*P* < 0.001) between nutrients, especially ammonium, urea and phosphate, and g*Noctiluca* abundance at both the Seeb Jetty and along the transect into the offshore waters of the Sea of Oman (Fig. S3). While algal blooms are usually stimulated by dissolved inorganic and dissolved organic nutrients from the environment [89], we have found that the major source of N and P nutrients for the development of the g*Noctiluca* bloom was in part derived from external uptake, but likely more from grazing on phytoplankton prey [9, 12]. Grazing is most likely an ancestral trait of *Noctiluca* as the exclusively heterotroph r*Noctiluca* is more prevalent in the world’s oceans and its elemental growth yield and excretion of N and P are highly affected by prey nutritional quality [90, 91]. In g*Noctiluca*, prey metabolism and nutrient uptake occur in tandem leading to the build-up of ammonium in the central cytoplasm of g*Noctiluca* [12], a process better studied in the heterotrophic r*Noctiluca*, which also has been shown to accumulate high concentrations of ammonium and excrete it into the surrounding waters [92–95]. Our transcriptomic data also indicated the active metabolism that generated ammonium. When released into the external environment, these nutrients could promote the population growth of its prey (e.g., diatoms). This recurrent process by which nutrients first accumulate in g*Noctiluca* cells primarily via grazing, and are then released into the ambient environment during the sexual reproduction phase, to be then used by diatoms or dinoflagellates, constitutes a positive feedback loop that can theoretically support g*Noctiluca* proliferation and bloom persistence over extended periods even under nutrient-poor conditions as in the cases of the Sea of Oman and the Arabian Sea.

The next step toward a bloom outbreak is a high level of cell proliferation and population growth. This appears to be achieved mainly through sexual reproduction. Specific growth rates attributable to asexual reproduction (binary division) of g*Noctiluca* are typically 0–0.28 d^−1^ [10], which would result in population growth of maximally 4 (=e^0.28*5^) folds in 5 days. This growth rate is far lower than 1.86 d^−1^, which occurred in the bloom event that we investigated. For sexual reproduction, g*Noctiluca* cell can generate 1024 gametes within a day [10], and typically every two gametes fuse to produce a trophont cell. This meiosis-driven reproduction can lead to an explosive growth and an abrupt bloom outbreak. Our transcriptomic data as well as microscopic images clearly show that this mode of reproduction is an essential part of the bloom formation. The potential of sexual reproduction to promote population (numeric) growth and bloom formation or sustenance has recently been proposed for other dinoflagellates as well [96].

The nutrient positive feedback loop can ensure an adequate supply of nitrogenous nutrients and energy that is essential for gamete production and outbreaks of g*Noctiluca* blooms. Although it has been reported that sexual reproduction in *Noctiluca* may be triggered by phagotrophic mode of nutrition [10, 49] and high density of ammonium [97], to this date, evidence in support of this notion has been circumstantial. Our transcriptome data, combined with the physiological results, provide insights that accumulation of ammonium derived primarily from metabolism of ingested prey might be responsible for inducing sexual reproduction in g*Noctiluca*, and the release of the gametes might accompany the release of the accumulated nutrients. Controlled experiments involving prey density and/or high ammonium concentrations in the growth medium will need to be undertaken to verify this possibility in the future. Furthermore, the transcriptomic evidence of enhanced photosynthesis yet depressed cell reproduction of *P. noctilucae in-hospite* leads us to hypothesize that g*Noctiluca* is capable of manipulating its endosymbiotic population (Fig. 4), enslaving them to be energy (ATP) and organic carbon providers. This additional source of energy that the host is able to obtain can potentially boost sexual reproduction accelerating bloom development. Similar host manipulation of symbionts has been reported in corals [82–84] and in the Acantharia-*Phaeocystis* symbiosis [98].

Although our field observations as well as transcriptomic analysis provide some valuable and insightful information about bloom formation in g*Noctiluca*, further studies involving metabolomics and advanced instrumentation such as nano-SIMS [99] or Single-Cell-Raman Scattering Microspectrometric Imaging [100] that allow probing of *in-hospite* host-symbiont C, N, P metabolite exchanges, will shed additional light on biochemical processes and mechanisms governing *gNoctiluca* bloom outbreaks. This information is important not only because there is growing evidence that g*Noctiluca* blooms may be expanding their range as a result of climate change and the spread of eutrophication and hypoxia in the global oceans, but also because of evidence showing that *gNoctiluca* blooms can: (1) short-circuit the food chain leading to the production of swarms of jellyfish and salps in place of fish, and (2) create water quality issues that impact freshwater, food, economic security and well-being of hundreds of millions of people living around impacted areas such as the Sea of Oman and the Arabian Sea.

## Supplementary information

Supplementary Information

## Data Availability

The datasets generated during and/or analyzed during the current study are available in the National Center for Biotechnology Information (NCBI) under the accession number PRJNA774540 (*gNoctiluca* and symbiotic *Protoeuglena noctilucae*) and PRJNA774510 (free-living *P. noctilucae*).
