# Supplementary material for: A grazing-driven positive nutrient feedback loop and active sexual reproduction underpin widespread Noctiluca green tides
Source: ISME Commun. 2022 Oct 20;2:103. doi: 10.1038/s43705-022-00187-4 (PMC9723592; doi:10.1038/s43705-022-00187-4)
Supplement: Supplementary file 1 — Supplementary Information [file 43705_2022_187_MOESM1_ESM.pdf]

A grazing-driven positive nutrient feedback loop and active sexual reproduction underpin widespread *Noctiluca* green tides

Running title: The drivers of green *Noctiluca* bloom

Hao Luo[1], Jingtian Wang[1], Joaquim I. Goes*[2], Helga do R. Gomes[2], Khalid Al-Hashmi[3], Craig Tobias[4], Claudia Koerting[4], Senjie Lin*[1,4]

This PDF file includes:

Figs. S1 to S10
Tables S1

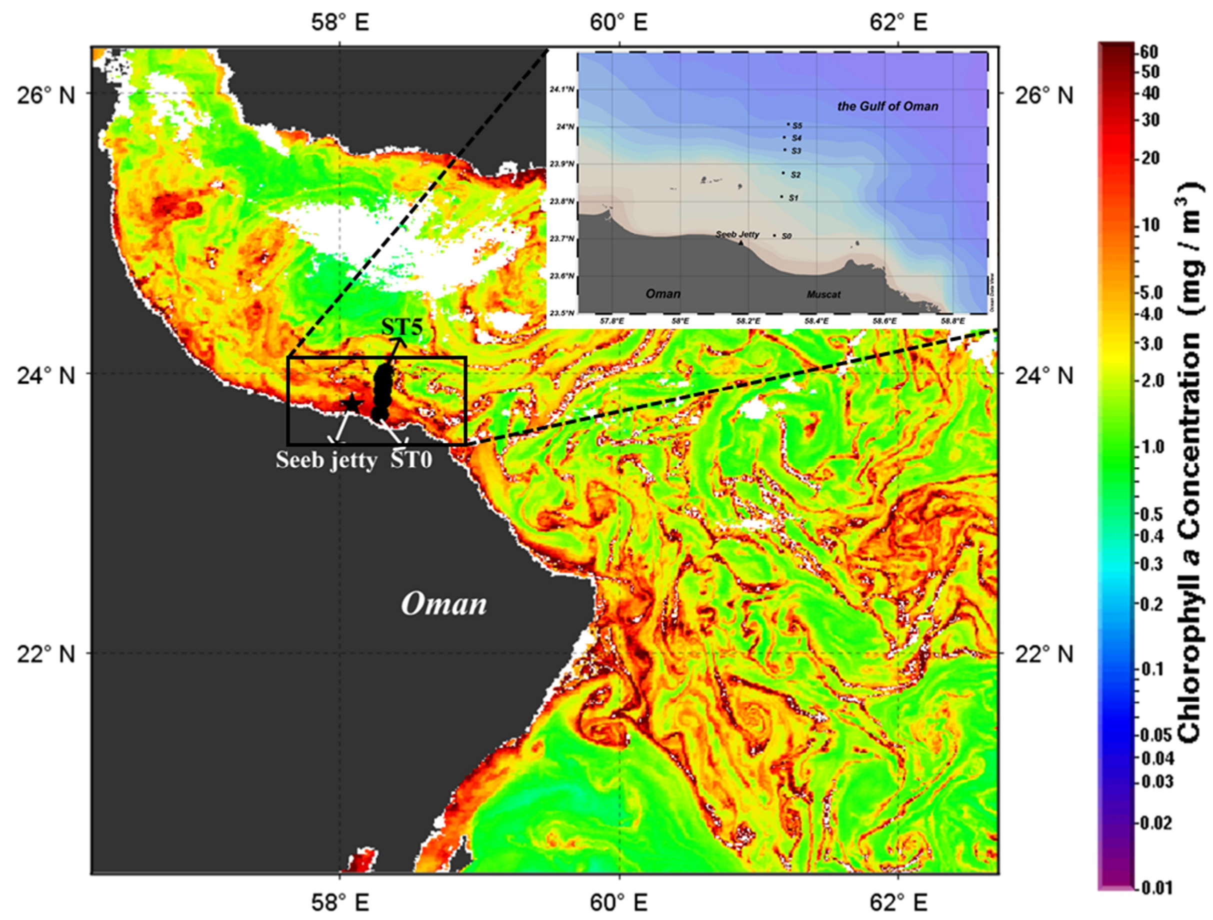

Fig. S1.

Primary sampling site at Seeb Jetty, Oman and sampling points (ST0-ST5) on a transect extending offshore into the Sea of Oman, overlaid on a MODIS-Aqua Level 2 Chlorophyll-a map for the 8th of February 2018.

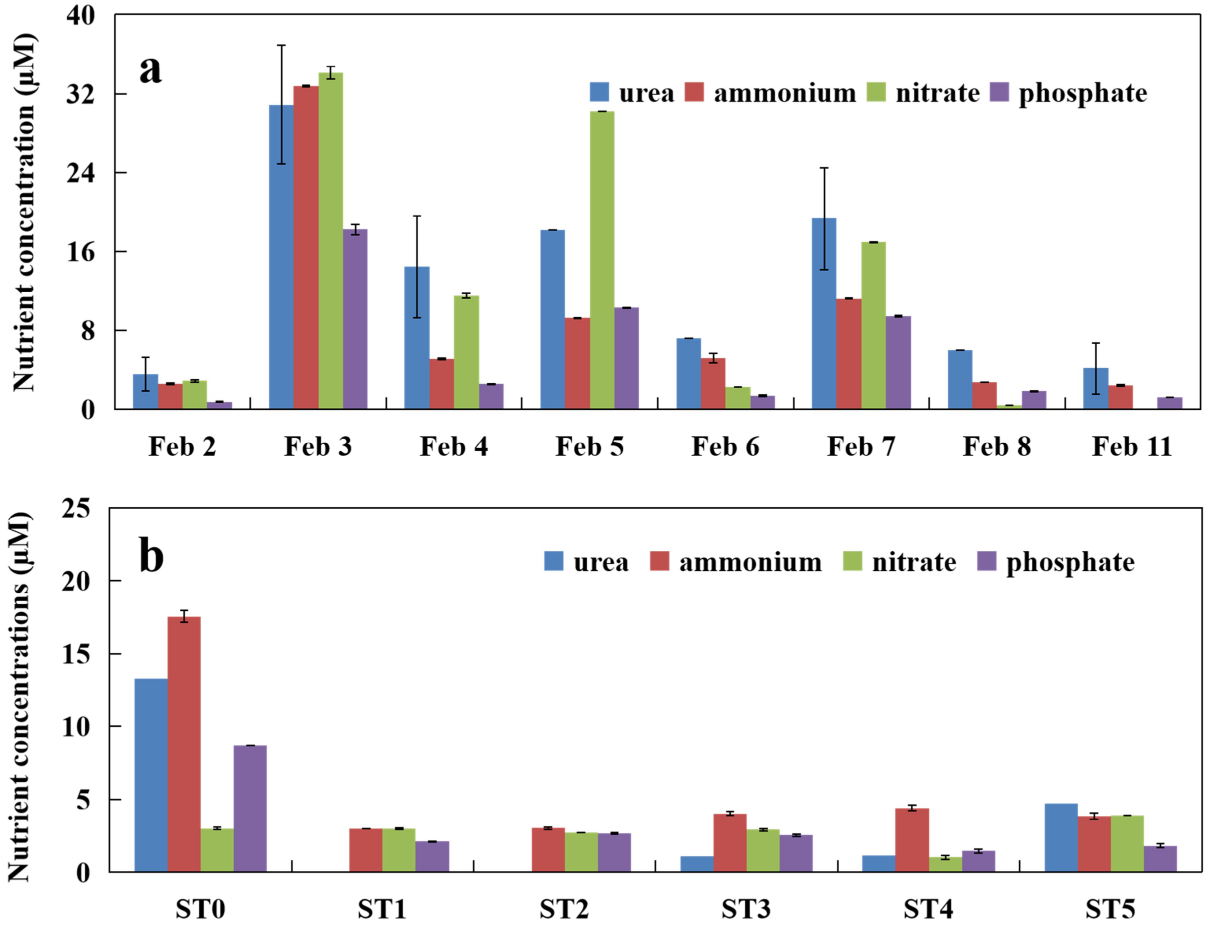

Fig. S2.

Nutrient concentrations (µM) monitored during the investigation. a, Seeb Jetty. b, along the 20 km transect from ST0 to ST5 in the Sea of Oman. Error bar stands for standard deviation. *gNoctiluca* bloom was observed at Seeb Jetty on 3 Feb as well as ST0 in the transect.

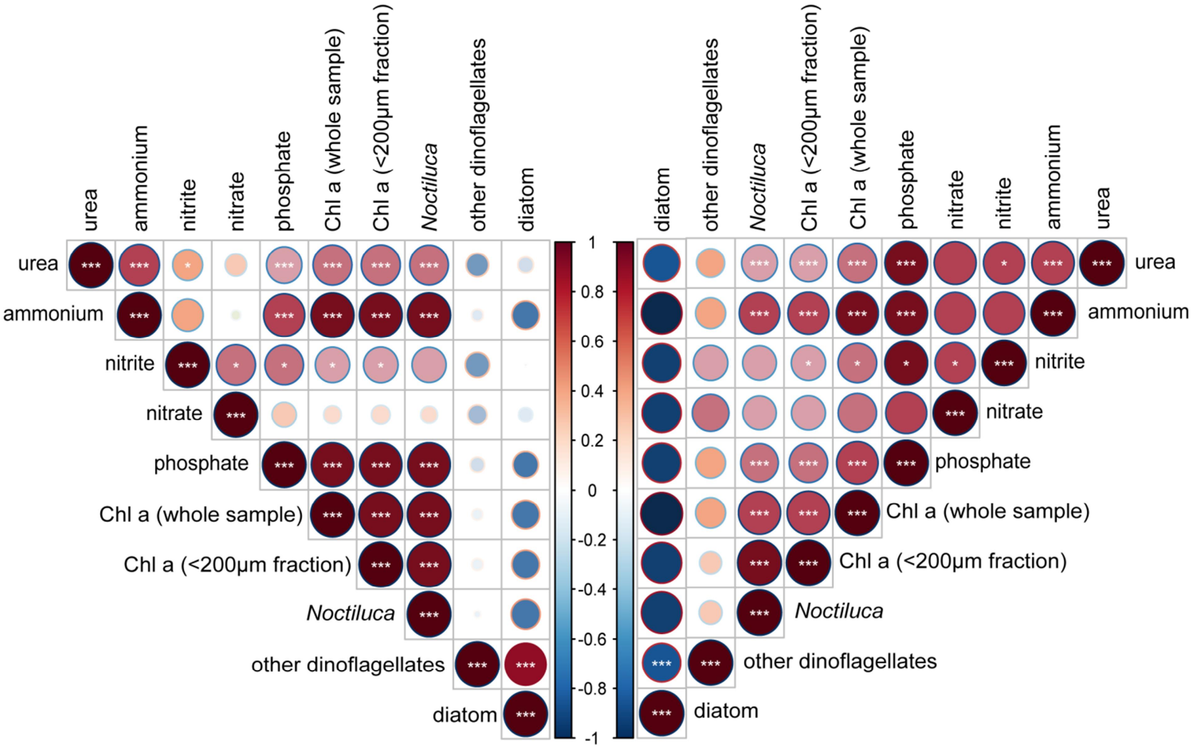

Fig. S3.

Correlation analysis between *gNoctiluca*, phytoplankton populations and various environment factors at Seeb Jetty (right) and at stations in Sea of Oman (left). Red stands for positive and blue stands for negative correlation. Circles with asterisks indicate significant differences (***P < 0.001).

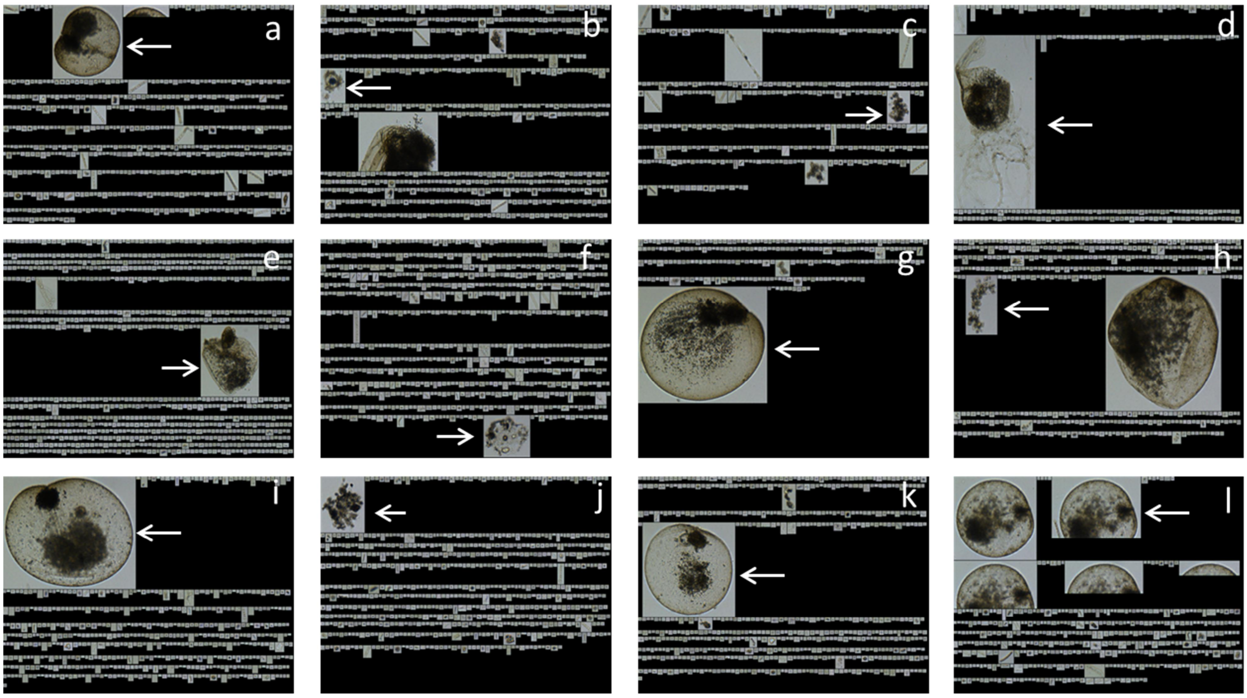

Fig. S4.

FlowCAM images of samples at Seeb Jetty on Feb 2 (a, b and c), Feb 3 (d and e), Feb 4 (f), Feb 5 (g and h), Feb 6 (i and j), Feb 7 (k) and Feb 8 (l). Among these, a, g, i, k and l (arrows) show the intact *gNoctiluca* cells with endosymbionts. b and f (arrows) show ghost cells after most gametes have been released. c, h and j (arrows) show dense gametes presented as clumps. d and h (arrows) show the *gNoctilcua* fragments. (Magnification: a-l 40x).

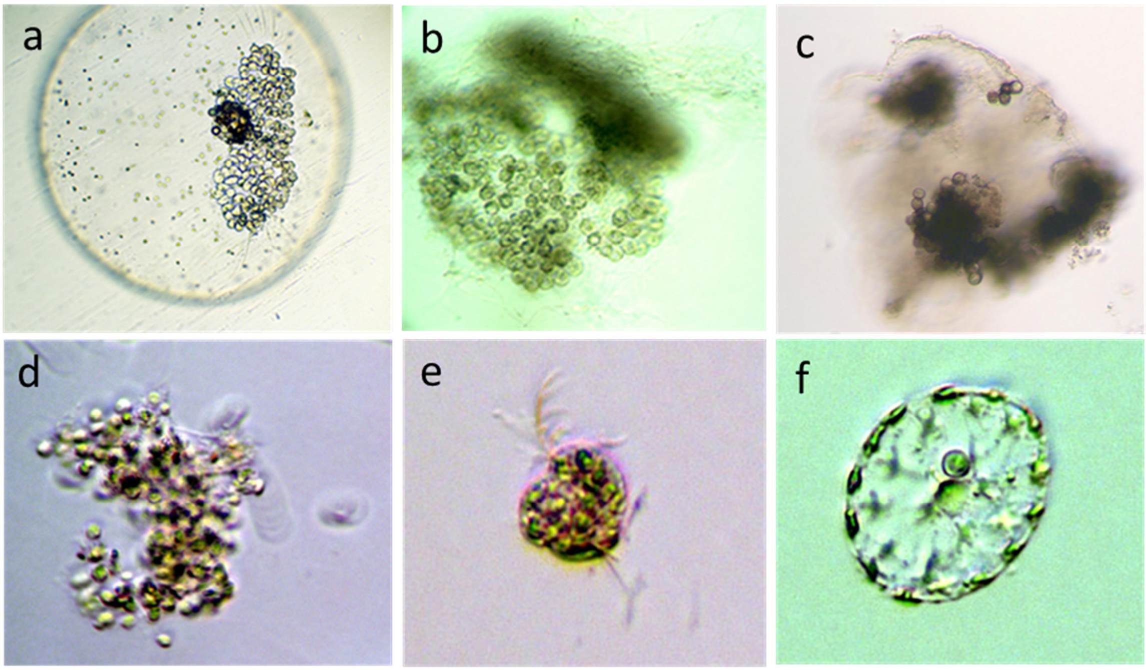

Fig. S5.

Microscopic images of samples at Seeb Jetty on Feb 2. a, *gNoctiluca* parent cell with progametes. b, c and d, clumps and cell fragments showing gametes. e and f, zygote and young *gNoctiluca*. (Magnification: a-f 40x).

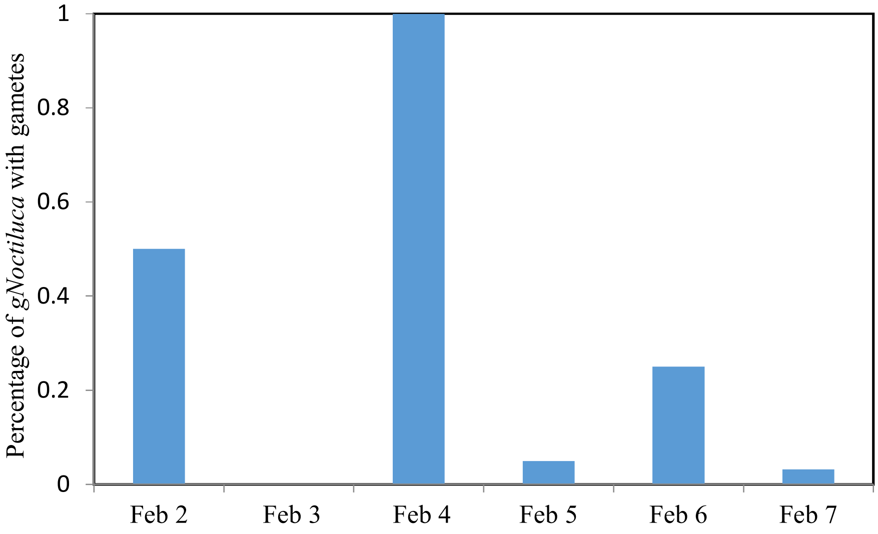

Fig. S6.
Percentage of *gNoctiluca* cells with gametes at Seeb Jetty during the bloom by FlowCAM images.

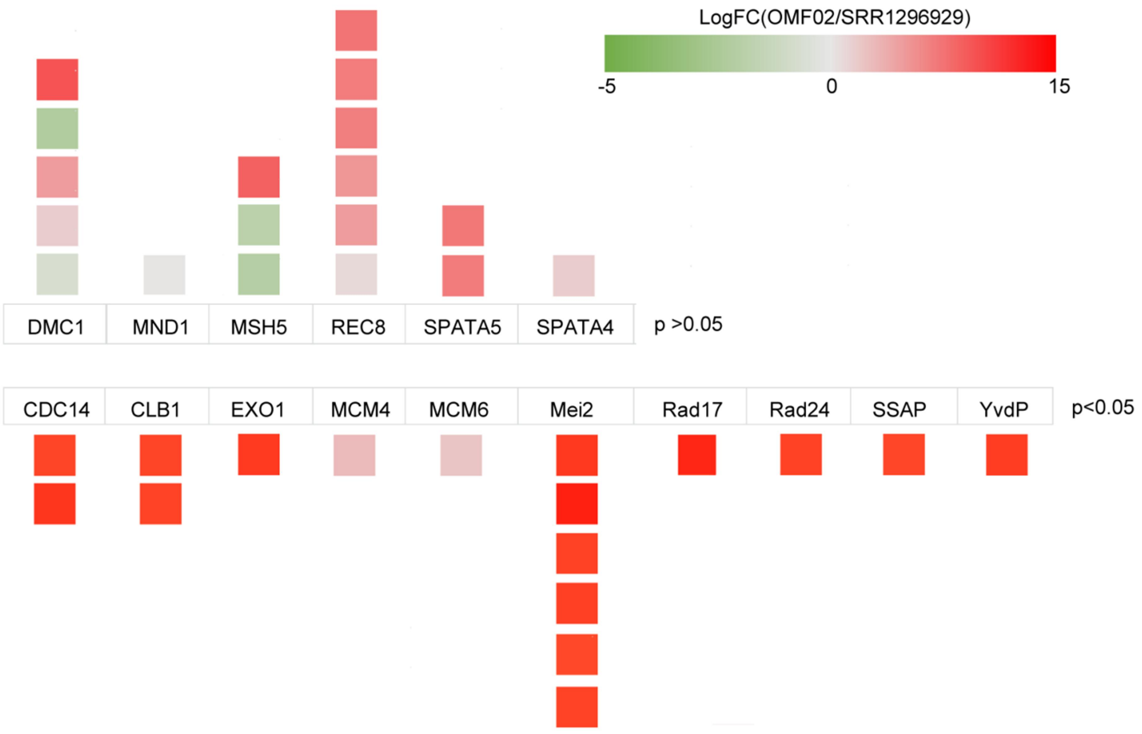

Fig. S7.

Heatmap of upregulation (red) and downregulation (green) of meiosis genes in g*Noctiluca* bloom relative to cultured *Noctiluca*. Each square represents a unique gene in the gene family, the function of which is indicated on the top or bottom of the stack of squares. Color scale denotes gene expression level in our sample (OMF02) relative to cultured *Noctiluca* sample (SRR1296929).

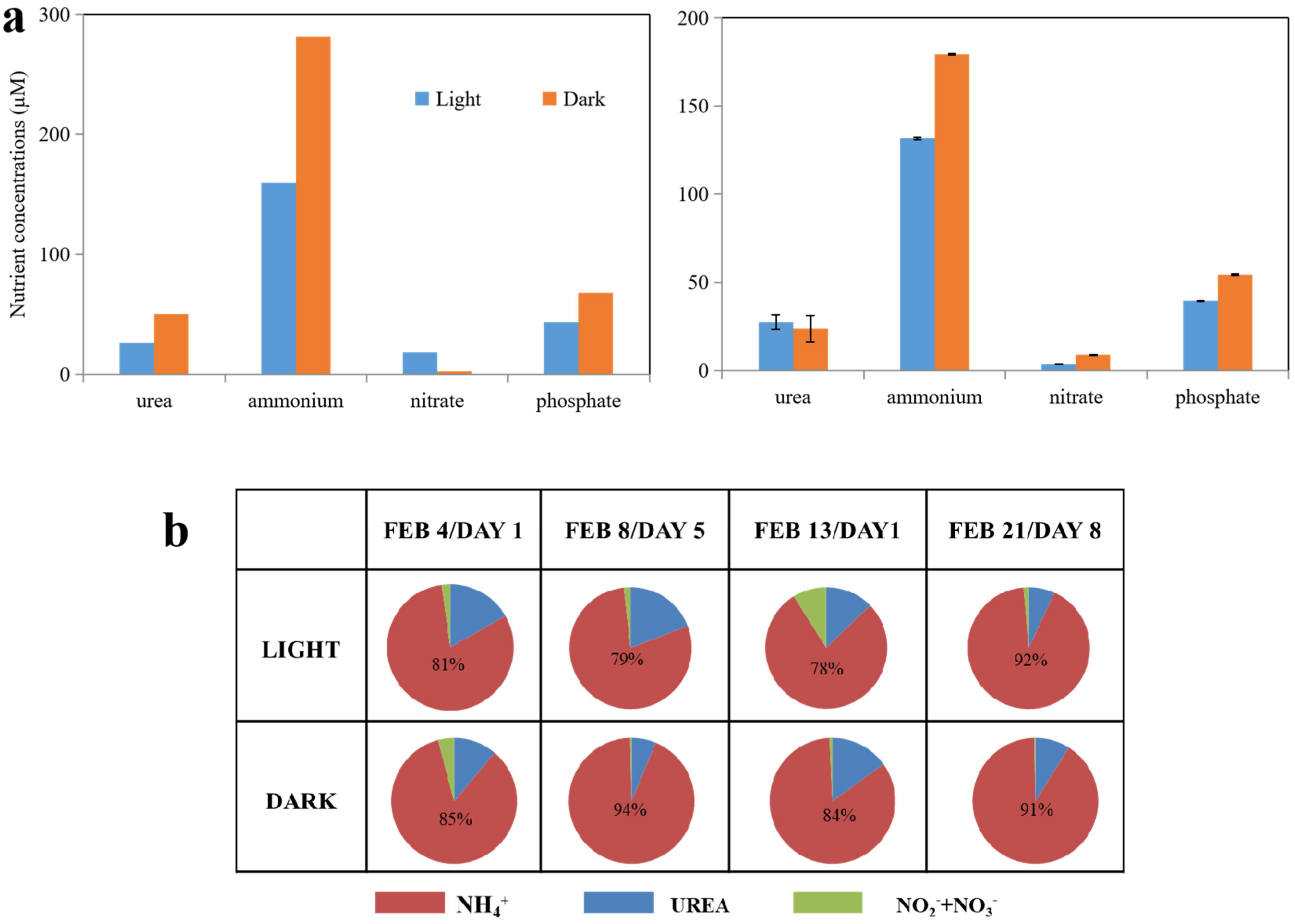

Fig. S8.

Nutrient release from isolated *gNoctiluca* cells incubated in FSW. a, urea, ammonium, nitrate and phosphate concentrations in dual experiments of g*Noctiluca* incubation. Left: *gNoctiluca* isolated from 3 Feb. 2018. Right: *gNoctiluca* isolated from 13 Feb. b, percent nitrogenous nutrients, ammonium, urea and nitrite+nitrate from the Seeb Jetty bloom (4 and 8 Feb) and from the ST0 bloom (13 and 21 Feb).

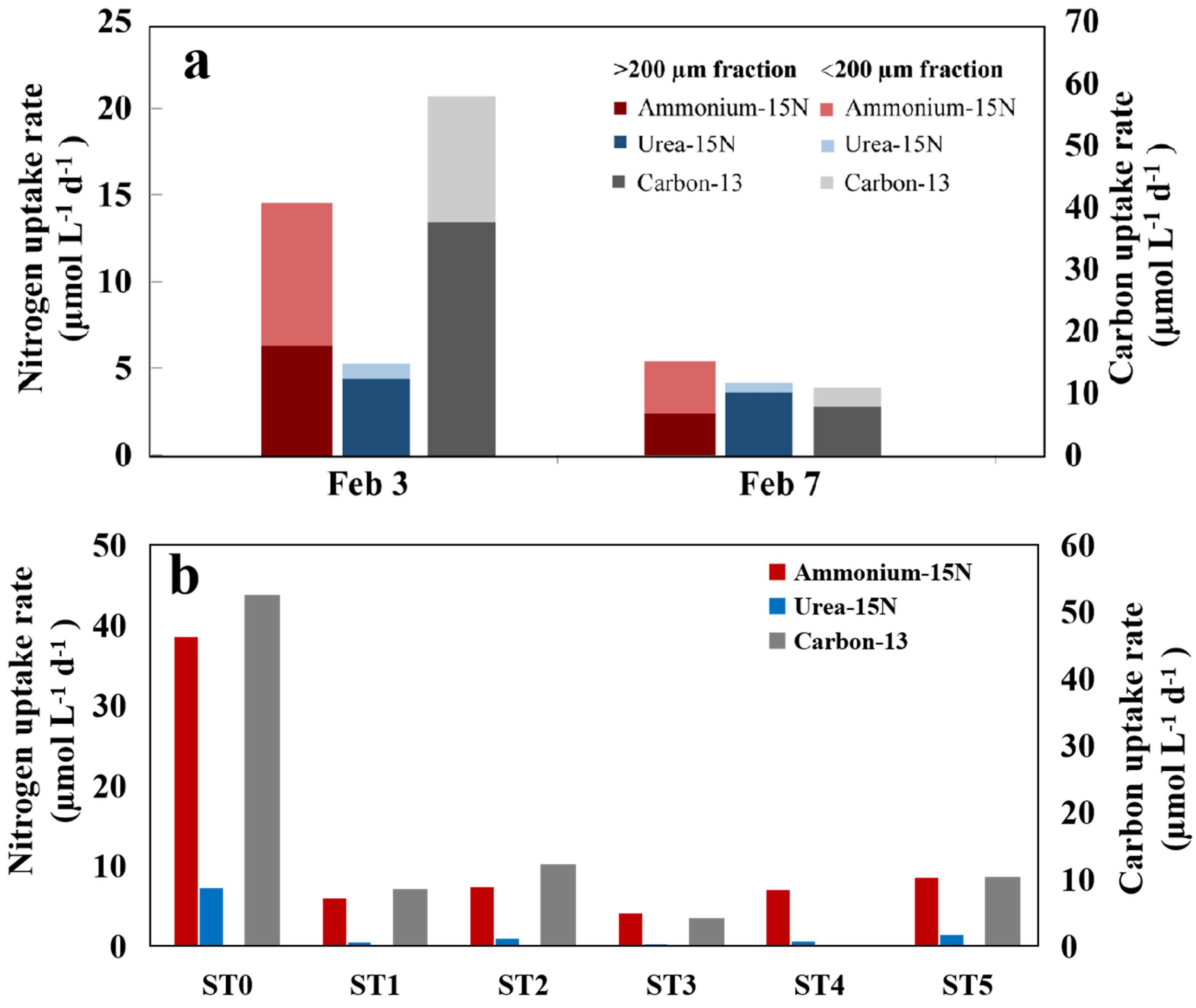

Fig. S9.

Uptake rate of nitrogenous nutrients ($NH4^+$ and urea; µmol L^{-1} d^{-1}) and inorganic carbon measured using ^{15}N and ^{13}C tracer, respectively. a, samples (>200 µm fraction (*gNoctiluca*) and <200 µm fraction) from Seeb Jetty. The >200 µm fraction contained only *gNoctiluca* whereas the <200 µm contained other protists with some small-sized *gNoctiluca*; therefore, *gNoctiluca* uptake rates ranged from the level marked by the >200 µm (bottom, darker bar) to the top of the stack (>200 plus <200, i.e. whole water). b, samples from stations along the Sea of Oman transect, among which *gNoctiluca* was dominant at ST0. Left axis indicates ammonium/urea uptake rate and right axis indicates carbon uptake rate. Note that the uptake rates for both N and C were dramatically higher in the *gNoctiluca* bloom waters (Feb 3 at Seeb Jetty and ST0 at the transect) than non-bloom waters.

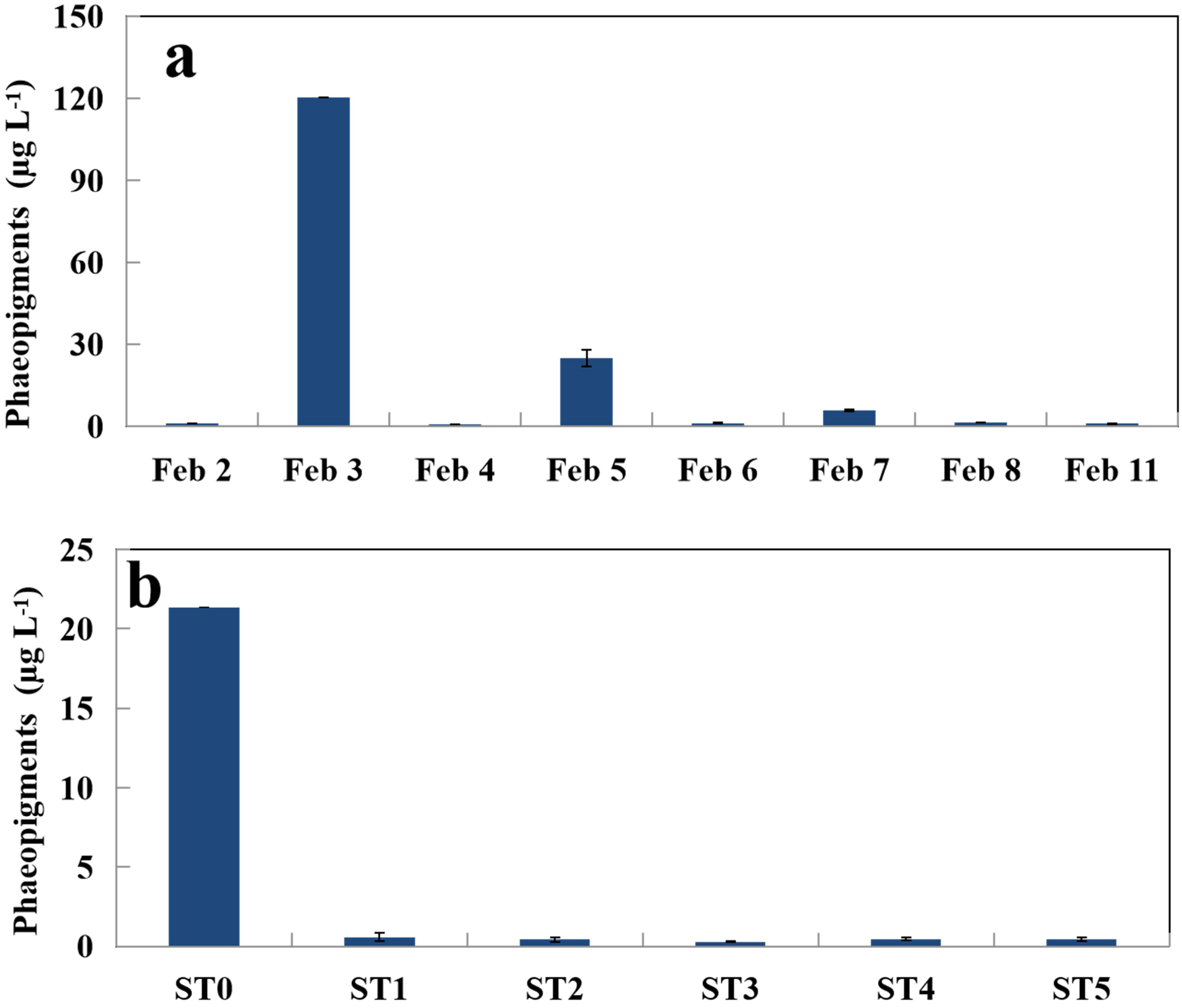

Fig. S10.

Concentration of phaeopigments, the digested products of chlorophylls ($\mu g\ L^{-1}$). a, Seeb Jetty. b, stations along the Sea of Oman transect. Error bar stands for standard deviation.

	Gene	FPKM		Gene	FPKM
Cytoskeleton, motility and particle uptake	Actin family	11449.29	Phagosome maturation	Syntaxin	213.31
	Dynein family	5067.59		Sec22	120.46
	Tubulin alpha	1097.34		Sec61	334.20
	Tubulin beta	3091.25		Rab5	23.57
	Kinesin family	1765.33		Rab7	93.87
	Myosin family	1177.65		PIK3C3	55.53
	Flagella-related protein	621.08		vATPase	479.86
	ARP 2/3 complex	110.25		PIKFYVE	48.56
	MRCK	19.82		TAP	240.19
	Calreticulin	770.35		NOS	49.56
	Calnexin	18.56	Digestion	Cathepsin family	21984.18

Table S1.
Expression profile of the phagocytosis pathway genes in the green *Noctiluca*.
